# Male Germ Cell Telomeres and Chemical Pollutants

**DOI:** 10.3390/biom13050745

**Published:** 2023-04-25

**Authors:** Gabriella Chieffi Baccari, Giuseppe Iurato, Alessandra Santillo, Brian Dale

**Affiliations:** 1 Dipartimento di Scienze e Tecnologie Ambientali, Biologiche e Farmaceutiche, Università della Campania “Luigi Vanvitelli”, 81100 Caserta, Italy; gabriella.chieffi@unicampania.it; 2 Ministero dell’Istruzione e del Merito, 00153 Roma, Italy; 3 Centro Fecondazione Assistita (CFA-Italia), 80127 Napoli, Italy; brian.dale@virgilio.it

**Keywords:** telomere, male germ cells, sperm, pollutants, fertility, reproduction, spermatogenesis

## Abstract

In recent decades, male infertility has been correlated with the shortening of sperm telomeres. Telomeres regulate the reproductive lifespan by mediating the synapsis and homologous recombination of chromosomes during gametogenesis. They are composed of thousands of hexanucleotide DNA repeats (TTAGGG) that are coupled to specialized shelterin complex proteins and non-coding RNAs. Telomerase activity in male germ cells ensures that the telomere length is maintained at maximum levels during spermatogenesis, despite telomere shortening due to DNA replication or other genotoxic factors such as environmental pollutants. An emerging body of evidence has associated an exposure to pollutants with male infertility. Although telomeric DNA may be one of the important targets of environmental pollutants, only a few authors have considered it as a conventional parameter for sperm function. The aim of this review is to provide comprehensive and up-to-date data on the research carried out so far on the structure/function of telomeres in spermatogenesis and the influence of environmental pollutants on their functionality. The link between pollutant-induced oxidative stress and telomere length in germ cells is discussed.

## 1. Introduction

Successful reproduction is fundamental for human society. However, dyscrasias exist between reproductive efficiency and one’s lifespan as reproductive organs age due to their dependence on sexual hormone levels, which shield health via their action on multiple organs in the human body. These hormone levels often fluctuate along a lifespan, which may lead to organismal decay and infertility when they decrease. Thus, an increase in age in males has a significant negative impact on testicular function, sperm functionality and, consequently, fertilization [1,2]. Gamete quality is also impaired by an imbalance in the redox status [3] and the continuous accumulation of genetic mutations [4,5], including Y-chromosome microdeletions [6] and DNA alterations [7] with reduced repair capacity [8]. It is widely accepted that telomeres are involved in biological ageing. Telomeres are ribonucleoprotein structures that are located at the end of all eukaryotic chromosomes and are involved in maintaining genomic integrity and stability [9]. In vertebrates, telomeres are composed of thousands of hexanucleotide DNA repeats (TTAGGG) that are coupled to specialized shelterin complex proteins and non-coding RNAs [10]. Although the telomere sequence is conserved among mammals, different tissues and cells within the same individual have varying telomere lengths [11]. In somatic cells, the telomere length shortens following each DNA replication cycle, but also in response to genotoxic insults and/or reactive oxygen species. The hypothesis that telomere shortening during successive cell divisions is a factor in aging was first proposed by Olovnikov in 1971 [12,13]. At that time, this idea seemed fantastic, but later the scientists involved in this discovery received the Nobel Prize [14,15,16]; today, this science is the basis for a new research in longevity. One peculiarity of germ cells, stem cells, granulosa cells and early embryos, is that they express telomerase, an enzyme, which has the potential to counteract the shortening of telomeres. Therefore, the presence of telomerase activity in male germ cells ensures that the telomere length is maintained at a maximum level during spermatogenesis, despite telomere shortening due to DNA replication or other genotoxic factors [17]. Spermatozoa, which are terminally differentiated cells, have longer telomeres than spermatogonia and pachytene spermatocytes. In addition to genetic factors and lifestyle factors, psychological stress also plays a crucial role in modulating the telomere length. An emerging body of evidence has associated the exposure to pollutants with male infertility. Telomeric DNA may be one of the important targets of environmental pollutants. Many epigenetic changes occur during spermatogenesis [18,19]. Above all, in mature sperm, the testis-specific histone H2B protein variant has been identified, which has a truly unique expression. Indeed, it does not undergo polyadenylation at its 3′ terminal, and it is localized in the telomeres of sperm chromosomes; furthermore, it seems to have telomere-associated functions and participates in the telomere-binding complex in the human sperm [20]. Therefore, environmental pollutants could have an impact on the epigenome of spermatozoa, consequently affecting male fertility. To date, there is little information on the association between environmental pollutants and telomere in male germ cells and the underlying molecular mechanisms that influence their structure/function.

As such, the aim of this review is to provide comprehensive and updated data on the importance of telomeres in spermatogenesis and the influence of environmental pollutants on their structure/function. The link between pollutants-induced oxidative stress and telomeres in germ cells is discussed.

## 2. Telomeres and Spermatogenesis

In recent decades, telomere biology has become an important topic in the field of human reproduction, prompting studies and research that are aimed at investigating the role of telomere length in spermatogenesis and male fertility, as well the development and quality of embryos during assisted reproduction treatments. The studies conducted so far on the topic are mainly clinical–epidemiologic, whereas biomolecular research on this aspect is lacking, leaving open the biological explanation that links telomere length and male infertility.

Telomeres regulate the reproductive lifespan by mediating synapsis and the homologous recombination of chromosomes during gametogenesis [21]. Consequently, disruptions to the balance and stability of the telomere complex (i.e., telomere homeostasis) in the germline has also been correlated with male and female infertility [17,21,22]. Short telomeres result in meiosis arrest, segregation and disjunction abnormalities, leading to the increased production of aneuploid germ cells [21]. Furthermore, short telomeres in men cause germ cell apoptosis, while in women, they cause meiotic arrest. Some telomere biology theories that are related to reproductive ageing regard telomere shortening in the female germ line as one of the main causes of reproductive ageing in women (for more on this topic, refer to specific reviews [23,24,25]). In contrast, in men, spermatogonia can rejuvenate telomere reserves throughout life via the activity of telomerase, the enzyme responsible for the maintenance of the length of telomeres [26].

**Figure 1:**
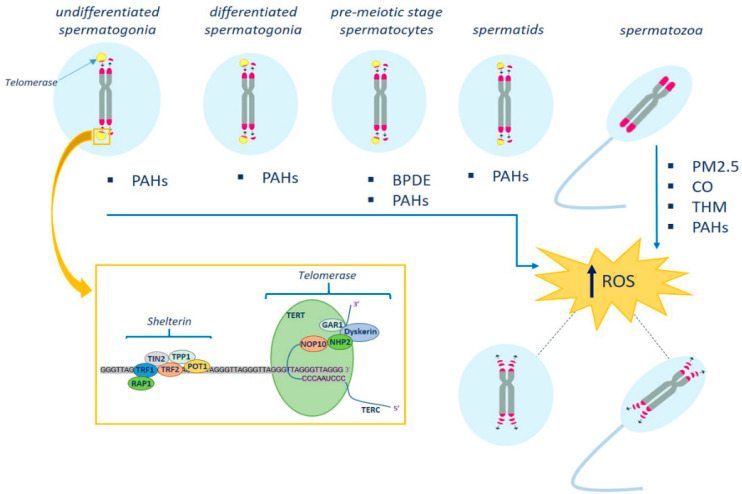
Schematic representation of the effects of environmental pollutants on telomere structure in male germ cells. Telomerase activity ensures that telomere length is maintained at maximum levels during spermatogenesis. Telomerase levels are high in undifferentiated spermatogonia, and progressively decrease in spermatocytes and spermatids until inactivation in mature spermatozoa. The latter have the longest telomeres. Environmental pollutants, by increasing oxidative stress, may act on germ cell telomere length and telomere-associated proteins. In the box are shown the telomerase and shelterin complexes (see text for details). PM_2.5_, particulate matter; THM, trihalomethanes; PAHs, polycyclic aromatic hydrocarbons; BPDE, benzo[*a*]pyrene—dihydrodiol-9,10-epoxide.

In addition to shelterin (consisting of six proteins, TRF1, TRF2, RAP1, POT1, TPP1, and TIN2) which protects telomeres and regulates telomerase, the telomere structure is complemented by other elements (Figure 1). They are the cohesins and telomerase complexes, including telomerase reverse transcriptase (TERT, the catalytic subunit of telomerase), the RNA component (TERC), and the dyskerin protein complex (dyskerin, NOP10, NHP2, and GAR1). TERT adds new telomeres (TTAGGG repeats) onto the chromosome end by using the template provided by TERC (Figure 1). 

A recent study using TERT reporter mice revealed that a high level of telomerase expression is a hallmark of undifferentiated spermatogonia, the mitotic population in which germline stem cells reside [26]. Telomerase levels in undifferentiated spermatogonia are comparable to those in embryonic stem cells and are much higher than in somatic progenitor compartments. Telomere shortening in telomerase knockout strains causes the depletion of undifferentiated spermatogonia and the final loss of all germ cells after undifferentiated spermatogonia fall below a critical threshold [26]. In spermatocytes and spermatids, telomerase activity progressively decreases until it reaches complete inactivation in mature spermatozoa [27,28]. Therefore, understanding the regulation of telomere structure and function is of paramount importance to identify the factors involved in the generation of infertile phenotypes.

In this regard, numerous studies in humans have shown that the telomere length in ejaculated sperm may be associated with male reproductive disorders [29,30], and that telomere shortening could be related to the production of sperm with morphological alterations, chromosomal rearrangements, characteristics of immaturity and elevated DNA damage [31,32,33]. Studies indicate that telomerase activity and human TERT (hTERT) expression are lower in infertile males, indicating a close association between telomere regulation and male sterility [34]. Decreased levels of both telomeric repeat-containing RNA (TERRA) and telomerase, and an altered telomere–TERRA association, have been detected in primary spermatocytes in infertile patients compared to control individuals [22,35]. However, with regard to the relationship between telomere length and clinical outcomes, the data in the literature are conflicting [36,37]. On the one hand, studies in infertile men have shown that patients with short telomeres are unable or less likely to produce good-quality embryos compared to those with longer telomeres [23,33,38]. On the other hand, however, other authors have found no statistically significant differences in reproductive outcomes between spermatozoa with long and short telomeres and donor samples [39,40,41]. Although epidemiological retrospective studies can provide insight into general trends regarding the association between telomeres and infertility factors, they have limitations; these can include the sample size not always being sufficient to validate statistical results. An accurate recent meta-analysis [42] that included all experimental studies exploring the association between sperm telomere length (STL) and male semen quality, male infertility and embryonic development, proposed that STL might be considered a good diagnostic marker and have predictive value regarding male fertility and clinical pregnancy. Therefore, STL could be used as a new biomarker for diagnostic male infertility and could be useful, in particular, for identifying and selecting germ cells with the greatest fertilization potential [30].

Although the telomere length has attracted much interest in the field of human reproduction, the telomere length as a biomarker of sperm quality and as a marker of fertility has been studied in pigs [28]. The results show that the sperm telomere length can be a useful biomarker for embryonic development in pigs, as sperm with longer telomeres produce higher rates of morulae and blastocysts. It is noteworthy that the quality of the spermatozoa of boars is much better than that of healthy human sperm donors, which is revealed via ultrastructural examination and morphological parameters [43]. This may be due to the careful selection of boars in order to produce offspring in farm animals. 

## 3. Germ Cell Telomere and Environmental Pollutants

The effects of environmental pollution on sperm quality has been the subject of numerous epidemiological investigations, and several reviews have recently been published on the topic [44,45,46,47]. Although numerous authors have identified excessive telomere shortening in the somatic cells of subjects exposed to air pollutants [48,49,50], to date, studies analyzing the influence of environmental pollutants on telomere structure/function in germ cells are very limited. Indeed, the sperm parameters considered in the research are predominantly on sperm volume, sperm concentration, sperm count, motility and morphology, DNA fragmentation and chromatin integrity.

Studies that have focused on gaseous air pollutants or particulate matter (PM) (PM_2.5_, PM_10_, SO_2_, NO_2_, CO and O_3_) have highlighted their negative correlations with sperm quality [46,51,52,53,54,55]. Significant alterations in at least one of the sperm parameters in association with at least one of the pollutants studied were detected. In particular, the sperm volume and total sperm count were found to be significantly negatively associated with smoking, carbon disulfide and traffic pollution [47]. Traffic pollution included common gaseous pollutants such as nitrogen oxides, sulfur compounds, and sulfur oxides. However, the sperm volume and total sperm count were not significantly influenced by lead exposure and environmental pollution. The latter included sulfur dioxide, nitric anhydride, nitrogen oxides and sulfur oxides, sulfur dioxide, nitrogen oxides, carbon monoxide, ozone, methane, non-methane hydrocarbons and volatile organic compounds [47].

Huang and collaborators [46], investigating a cohort of approximately 1100 men, provided evidence that exposure to PM_2.5_ and its constituents (antimony, cadmium, lead, manganese and nickel) may contribute to decreased semen quality. The daily average concentrations of PM_2.5_ constituents were continuously collected at fixed monitoring stations that were away from industrial sources, traffic, buildings, or residential resources of emissions, such as the burning of coal, waste or oil. The authors used three different statistical models to estimate the associations between PM_2.5_ constituent exposures and semen quality. The study demonstrated that each increase in the interquartile range (36.5 μg/m^3^) of PM_2.5_ exposure was significantly associated with an approximately 8% decrease in sperm concentration (95% CI: 2.3%, 14.4%) and in total sperm count (95% CI: 0.7%, 15.0%). Exposure to antimony, cadmium, lead, manganese and nickel was significantly associated with a decrease in sperm concentration, while exposure to manganese was also significantly associated with a decrease in total motility. Non-smokers were more sensitive to exposure to PM_2.5_ constituents, particularly antimony and cadmium. However, although the study was carried out on relatively large population, it presents several limitations. Firstly, the monitoring data came from only two fixed stations and did not take into account spatial variations; secondly, other air pollutants, including ozone, carbon monoxide, etc., were not considered; thirdly, most subjects underwent only one semen examination; and lastly, the association between PM_2.5_ constituents and sperm morphology were not analyzed. It was also demonstrated that cadmium, a major constituent of PM_2.5_, could also induce altered spermatogenesis [56]. These metals can induce lipid peroxidation and testicular necrosis and apoptosis, which have been related to altered circulating androgen levels and fertility [46]. In conclusion, these studies suggest that gaseous air pollutants and PM may negatively affect sperm quality, particularly the sperm concentration.

Another recent review provides a solid summary of the existing works that correlate exposure to polycyclic aromatic hydrocarbons (PAHs) with male infertility [57]. PAHs are a large family that contain the most widespread environmental contaminants in the world. Usually, they attach to the surface of PM and can be absorbed through the skin, respiratory tract and gastrointestinal tract. The biomonitoring of levels of urinary PAH metabolites is an important approach used to measure human exposure and the burden of PAHs on the body [58]. The data showed that there is a significant negative relationship between PAH metabolites and sperm volume, concentration, motility, morphology, as well as an observed DNA degeneration. It is of note that the review emphasizes that the CYP1A1 genotype polymorphisms are more common in infertile men.

Endocrine disruptors (EDCs) in the environment are responsible for a decline in semen quality that has been most notable in the last few decades. A recent review provides results from epidemiological investigations over the last 30 years concerning the association between exposure to environmental and occupational pesticides (organophosphate, organochlorine, pyrethroid, carbamate, and other pesticide chemical groups) and semen quality [59]. The studies show that exposure to non-persistent EDCs, such as bisphenol A, triclosan, parabens, synthetic pyrethroids, organophosphate pesticides and phthalates, may decrease semen quality by affecting semen quality parameters such as sperm volume, total sperm count, motility, total motile count, sperm motion, sperm DNA damage (comet extent, tail length, tail distributed moment, percent of DNA located in the tail, DNA fragmentation index, high DNA stainability), the X:Y ratio and aneuploidy [45,59].

Finally, there has been a recent interest in electronic-waste-recycling-associated chemical exposure and intermediate health outcomes, including DNA damage. Significantly higher levels of DNA damage in spermatozoa, resulting in an increased risk of infertility, has been found in workers exposed to e-waste that contains harmful substances, including clastogens and aneugens [60].

Although the pathophysiology is not entirely clear, evidence from in vitro studies suggests that environmental pollutants may exert their toxic effect by acting directly on plasma membrane fluidity and the electrochemical potential of spermatozoa [61]. Pollutants may also act negatively on sperm motility by activating the apoptotic cascade [62] or by altering mitochondrial function and regulating pro-apoptotic genes at the mitochondrial level [63]. Finally, pollutants have been found to increase oxidative stress by increasing the production of reactive oxygen species, resulting in increased lipid peroxidation [63,64].

Thus, in general, exposure to environmental pollutants is a risk factor for impaired sperm quality. Although the affected parameters have varied among different studies, there is a general consensus that DNA damage is consistently present in human sperm. However, in none of the above papers is “telomere length” considered among the parameters assessed for sperm quality, which could add information on the possible DNA damage that is induced by an increase in the pressure of environmental pollution. In Table 1, we have reported the effects of pollutants on telomere length and telomere-associated proteins in male germ cells. In a recent study, 423 men were exposed to known concentrations of PM_2.5_, PM_10_, CO, SO_2_, NO_2_ and O_3_ in order to evaluate the STL [65]. The results show a negative association between CO and PM_2.5_ and STL (Table 1). However, the results of this study are in contrast to the results obtained from a pilot biomonitoring study carried out on a smaller sample of normospermic men (112 men) that were clinically healthy and resided in areas with high or low environmental pressure [66]. Through quantitative real-time PCR, the authors demonstrated that STL was significantly longer in the high group than in the low group. It should be underlined that (1) the relatively small sample size and (2) lack of pollutant monitoring represented the fundamental limitations of this study.

An association between trihalomethane (THM) exposure and telomere dysfunction in sperm has recently been demonstrated [67]. In particular, inverse associations were found between concentrations of chloroform (TCM), Br-trialomethanes (Br-THM), bromodichloromethane (BDCM), bromochloromethane (DBCM) and bromoform (TBM), and the TL of spermatozoa (Table 1).

As mentioned above, PAHs can cause reproductive toxicity. One study evaluated the potential association between PAH exposure and human STL [68]. Data indicate a correlation between the urinary levels of 1-hydroxypyrene (1-OHPyr) and 1-hydroxynapthalene (1-OHNap) and telomeric DNA damage in sperm. The results were confirmed by in vivo animal experiments showing that administering benzo[*a*]pyrene (B[*a*]P) (a ubiquitous PAH pollutant) to rats for 4 weeks causes a shortening of the STL and a decrease in the TERT expression in germ cells in a dose-dependent manner [68] (Table 1). In conclusion, environmental exposure to some PAHs may be associated with STL shortening in humans. In addition, in vivo animal studies also demonstrate the negative effects of B[*a*]P on male germ cell telomeres. In a later study, Ling and collaborators [69] explored the effects of benzo[*a*]pyrene—dihydrodiol-9,10-epoxide (BPDE), the active metabolite of B[*a*]P, on telomere dysfunction in mouse spermatocyte-derived cells (GC-2), and also the potential role of telomerase in BPDE-induced sperm cell damage. The results showed that BPDE induced the inhibition of cell viability, senescence and apoptosis in GC-2 cells in a dose-dependent manner. Telomere shortening, telomere-associated DNA damage, decreased TERT expression and telomerase activity, and the activation of the DNA damage response pathway (ATM/Chk1/p53/p21) were also observed in BPDE-treated cells (Table 1). Furthermore, in cellular models of TERT knockdown and re-expression, it was shown that TERT regulated the telomere length and the expression of DNA damage response-related proteins, thus influencing senescence and apoptosis in GC-2 cells. These in vitro results were further confirmed in vivo in the testicular cells of rats that were orally administered B[*a*]P for 7 days. Treatment with B[*a*]P resulted in histological lesions, apoptosis and senescence in rat testes, accompanied by telomere shortening, reduced TERT protein levels and an increased expression of DNA damage response proteins (Table 1). In conclusion, TERT-mediated telomere dysfunction contributes to B[*a*]P- and BPDE-induced senescence and apoptosis through the DNA damage response in male reproductive cells.

Finally, telomere dysfunction was found in rats treated with diphenylether decabrominate (BDE-209) and decabromodiphenylethane (DBDPE), which are common flame retardants used in many types of electronic and textile products [70]. Due to their persistence and bioaccumulation, BDE-209 and DBDPE are widely present in the environment and in wild animals. BDE-209 and DBDPE exposure induce an increase in oxidative stress and impair telomere function by shortening the telomere length and reducing telomerase activity (Table 1), resulting in cellular senescence and apoptosis in the rat testis [70].

In conclusion, a growing body of evidence suggests that telomere length is a sentinel biomarker of environmental exposure. The introduction of STL among the conventional parameters of sperm quality assessment could provide insight regarding the molecular mechanisms that underlie the alterations in the spermatozoa that are induced by environmental pollutants.

## 4. Germ Cell Telomere and Oxidative Stress

It has well known that reactive oxygen species (ROS) are required for the maintenance of mammalian spermatogonial stem cells (SSCs) and that a high glycolysis level favors the long-term self-renewal of SSCs [71,72]. In these cells, ROS were produced mainly by the ROS–BCL6B–NOX1 pathway and minimally by mitochondrial OXPHOS activity, which is much lower in SSCs compared to differentiating spermatogonia [72,73]. Unlike SSC self-renewal or spermatogonial proliferation, spermatogonial differentiation relies more on mitochondrial respiration. In fact, energy production is shifted from glycolysis to OXPHOS in spermatocytes and spermatids [73]. Spermatozoa utilize glycolysis for survival but require both glycolysis and OXPHOS for motility and fertilization. However, cellular ROS levels must be tightly regulated to maintain normal cell functions since excessive ROS production can cause oxidative damage, overwhelm the cellular antioxidant capacity and trigger apoptosis [74]. Indeed, the attachment of ROS to sperm DNA is believed to be a step in the cascade reaction that leads to DNA fragmentation and, ultimately, apoptosis [75,76,77]. Therefore, a delicate balance needs to be established in order to maximize the beneficial effects of ROS and prevent the detrimental effects of over-physiological levels.

It has been largely demonstrated that one of the toxic effects of pollutants is an increase in oxidative stress caused by an increase in the production of ROS [63,64]. ROS-induced oxidative stress is now recognized as the most common underlying mechanism that accelerates telomere shortening and dysfunction in somatic cells [78]. With regard to this, there is evidence that an increase in ROS can lead to alterations in the bases of DNA, such as the oxidation of guanine to 8-oxoguanine (8-oxoG), which ends up as an excised base [28,79]. The telomeric TTAGGG repeats show a high susceptibility to oxidative radicals and are sensitive to the accumulation of 8-oxoG, resulting in the alteration of telomeric proteins and the inhibition of telomerase, which in turn, results in the shortening, dysfunction and instability of telomeres [80]. ROS also induce single-strand breaks (SSB) in telomeres directly, leading to replication fork collapse and telomere loss [81]. Furthermore, the presence of the shelterin complex in telomeres prevents the recruitment of DNA damage response (DDR) proteins; therefore, damage to telomeres caused by ROS may not efficiently activate the DDR and hampers the DNA repair process downstream of the initial DDR [82]. ROS can also induce the disruption of the proteins that regulate telomere length, including telomeric repeat binding factor 1 and 2 (TRF1 and TRF2) binding [83,84]. Finally, evidence has indicated that the correspondence of oxidative adducts with the end of telomeric DNA prevents telomere lengthening [85].

Several enzymatic pathways are known to counter such oxidative insults. PRDX1 (peroxiredoxin 1, a ROS-scavenger) is highly enriched in telomeres during replication and reduces hydrogen peroxide to water and protects against oxidative attack [86]. In addition, 8-oxodG must be resolved by the base excision repair (BER) pathway. This starts with recognition and excision by 8-oxoG DNA glycosylase (OGG1), thus yielding an AP site, which must be cleaved by AP endonuclease 1 (APE1) and processed by downstream BER to restore the original G:C bp.

Therefore, it cannot be rejected that spermatozoa from a single individual could exhibit different telomere lengths not only due to specific telomerase activity during the early stages of spermatogenesis, but also due to their exposure to ROS and to the efficiency of the systems that counteract oxidative damage [29]. Spermatozoa have OGG1 and the capacity to excise 8-oxodG residues, and do not contain APE1 [87]. Furthermore, spermatozoa, due to their extremely reduced cytoplasm, have low amounts of antioxidant enzymes and consequently use the high antioxidant capacity of seminal plasma [88,89,90]. In the male urogenital tract, ROS mainly originate from leucocytes and abnormal immature spermatozoa. In moderate concentrations, ROS play an important role in post-testicular sperm maturation. They are involved in the formation of interprotamine disulphide bridges during epididymal transit, thus enhancing the nuclear condensation of spermatozoa [90]. ROS also participate in membrane tyrosine phosphorylation, which enables flagellar capacitation and hyperactivation [76,90]. In this regard, Mishra and collaborators [91] found longer telomeres in infertile men experiencing mild oxidative stress. Thus, although severe oxidative stress leads to extensive damage to biomolecules, a moderate oxidative stress level could be necessary for STL maintenance and beneficial to cellular homeostasis [37,90,91].

A recent prospective study investigated the relationship between oxidative stress and telomere attrition in the spermatozoa of infertile males with altered conventional seminal parameters by comparing them with the spermatozoa of males with normal conventional seminal parameters [90]. The results showed that in infertile males, increased oxidative stress is associated with impaired telomere interaction and chromatin condensation defects. Furthermore, the average number of telomeric signals per sperm was positively correlated with the percentage of sperm that show chromatin condensation defects. Accordingly, other studies have evidenced higher ROS levels in the spermatozoa of infertile men than in fertile men [37,92]. Consistently, an in vitro study showed that the addition of hydrogen peroxide, an effective ROS, to spermatozoa leads to a decrease in the STL, as measured by quantitative fluorescence during in situ hybridization (Q-FISH) [90,93]. Finally, an unhealthy lifestyle and behavioral habits such as smoking, alcohol consumption, nutrition and obesity can increase ROS production and negatively affect the STL [94,95]. Li and collaborators [70] found that exposure to persistent organic pollutants (BDE-209 and DBDPE) in rats led to an increase in oxidative stress, and induced the inhibition of telomerase activity with TERT downregulation. The molecular mechanism involved in how oxidative stress affects the telomere structure or telomerase activity remains to be clarified.

## 5. Conclusions and Perspectives

Evidence suggests that the altered structure and function of sperm telomeres is associated with male infertility. The few studies carried out to date, which are predominantly clinical–epidemiological, indicate that telomeric DNA may be one of the important targets of environmental pollutants, suggesting that “telomere length” is a sentinel biomarker of environmental exposure. Because chemical pollutants can trigger an increase in ROS in biological systems, we speculate that the induction of oxidative stress may be a possible mechanism that underlies pollutant-induced telomere dysfunction (Figure 1). Thus, the introduction of STL among the conventional parameters used for sperm quality assessment could expand our understanding of the link between pollutants and sperm quality. The extent and type of damage induced by chemical pollutants in germ cell telomeres during the proliferative and maturation processes remain to be clarified. Epidemiological studies on larger sample sizes that employ a molecular approach in order to investigate the mechanisms underlying the toxicity of environmental pollutants within the telomere complex need to be carried out.

## Figures and Tables

**Table 1:** Effects of pollutants on telomere lenght and telomere-associated proteins in male germ cells.

Pollutans		Sample	Damage	Refs
**CO**		Human sperm	Shorting STL	[65]
**PM_2.5_**		Human sperm	Shorting STL	[65]
**THM**	TCM, BDCM, DBCM, TBM	Human sperm	Shorting STL	[67]
**PAHs**	1-OHPyr, 1-OHNap B[*a*]P BPDE	Human sperm Rat testis Rat testis GC-2	Shorting STL Shorting germ cell TL; Telomerase inhibition; decrease in TERT protein expression Shorting TL; telomere-associated DNA damage; telomerase inhibition; decrease in TERT protein expression	[68] [68] [69] [69]
**BDE-209 DBDPE**		Rat testis	Shorting STL; telomerase inhibition	[70]

STL, sperm telomere length; CO, carbon monoxide; PM_2.5_, Particulate Matter 2.5; THM, trihalomethanes; TCM, chloroform; BDCM, bromodichloromethane; DBCM, dibromochloromethane; TBM, bromoform; PAHs, Polycyclic Aromatic Hydrocarbons; 1-OHPyr, 1-hydroxypyrene; 1-OHNap, 1-hydroxynapthalene; B[*a*]P, benzo[*a*]pyrene; BPDE, benzo[*a*]pyrene—dihydrodiol-9,10-epoxide; BDE-209, diphenylether decabrominate; DBDPE, decabromodiphenylethane.

## Data Availability

No new data were created or analyzed in this study. Data sharing is not applicable to this article.
